# The Unusual Suspect: Artery of Percheron Occlusion in a Young Healthy Patient

**DOI:** 10.7759/cureus.96953

**Published:** 2025-11-16

**Authors:** May Thet Htar Zon, Khine Wut Yee Lwin, Owoseni Owolabi, Siddiq Omer

**Affiliations:** 1 Stroke Medicine, Hereford County Hospital, Wye Valley NHS Trust, Hereford, GBR

**Keywords:** artery of percheron, bilateral thalamic infarction, clinical case report, patent foramen ovale (pfo), stroke in young population

## Abstract

The artery of Percheron (AOP) is a rare anatomical variant. It is a single arterial trunk that supplies the bilateral thalami and midbrain. It is the result of an anatomical variant of the diencephalic irrigation in which the thalamic paramedian arteries arise with a common trunk from the posterior cerebral artery. The AOP is associated with a clinical syndrome characterized by bilateral vertical gaze palsy, memory impairment, and hypersomnia. Occlusion of the AOP can result in bilateral thalamic infarction with highly variable presentations that often mimic conditions such as encephalitis or metabolic disorders. This case report aims to highlight the diagnostic challenge and clinical significance of recognizing AOP occlusion in young patients presenting with acute confusion. We report here a case of a 24-year-old woman who presented with acute confusion but no focal motor deficit. Initial management targeted viral encephalitis, but magnetic resonance imaging confirmed bilateral thalamic infarcts, and magnetic resonance angiography demonstrated left occlusion of the AOP. Further evaluations identified a large patent foramen ovale (PFO) as the likely embolic source. The patient recovered with cognitive rehabilitation and returned to work within two months. This case illustrates the importance of including AOP occlusion in the differential diagnosis of acute confusion in young adults. In addition, it reinforces the need to conduct a thorough etiologic assessment for stroke in young adults, particularly evaluating for cardioembolic mechanisms such as the PFO.

## Introduction

Strokes involving deep brain structures can be diagnostically challenging, particularly when they affect the thalami. The thalamus plays a central role in regulating consciousness, cognition, and sensory pathways, so lesions in this region often produce broad and nonspecific clinical manifestations. Patients may present with symptoms such as confusion, memory impairment, abnormal eye movements, and somnolence, which can closely mimic infectious, metabolic, or toxic encephalopathies [1]. This nonspecificity frequently results in diagnostic delays or misdiagnosis [1].

Infarction of the artery of Percheron (AOP) is an uncommon but important vascular cause of these symptoms. The AOP is a rare anatomical variant, first described by Percheron in 1973, in which a single arterial trunk supplies both paramedian thalami and, sometimes, the midbrain [2]. Occlusion of this artery produces a characteristic pattern of bilateral thalamic infarction [3] that accounts for a small fraction of ischemic strokes. Despite its rarity, early recognition is vital because timely neuroimaging and initiation of targeted management can significantly influence recovery.

This report describes the case of a young and otherwise healthy patient who developed bilateral thalamic infarction because of occlusion of the AOP. This case underscores two key clinical considerations: first, AOP infarction should be included in the differential diagnosis of acute confusion in young patients, particularly when initial evaluation does not reveal a clear metabolic or infectious source; and second, comprehensive evaluation for underlying embolic risk factors such as a patent foramen ovale (PFO) is essential when stroke occurs in this population, as such findings have important implications for secondary prevention [4].

## Case presentation

A 24-year-old woman who was working as a full-time veterinary doctor and participated in daily cross fitness exercises presented to the emergency department with acute disorientation, imbalance, lethargy, blurred vision, slurred speech, and light-headedness. Uncharacteristically, she was unable to recognize the time, place, or people around her. She reported an episode of sore throat one week before presenting to the emergency department. She had no history of recreational or performance-enhancing drug abuse, recent animal bites or scratches, or recent foreign travel.

An examination revealed global cognitive decline, with impairments in attention, orientation, memory, speech, abstract thinking, and calculation. She demonstrated no focal neurological deficit in the form of motor weakness, sensory impairment, or cranial nerve abnormalities, and there were no cerebellar signs. Her speech was confused, but there was no obvious dysphasia. Her scores on the Oxford Cognitive Screen were relatively low, with the most significant deficits observed in the domains of attention and recall. No neck rigidity or other sign of meningeal irritation was observed. Her cardiovascular, respiratory, and abdominal examinations were unremarkable, and there were no signs of infection.

The initial lab workup, including full blood count, C-reactive protein, renal, liver, and thyroid functions, and coagulation profile, was unremarkable. A computed tomography scan of the brain did not reveal any acute abnormality. Given the patient's presentation, magnetic resonance imaging (MRI) of the head and magnetic resonance angiography (MRA) were requested, a lumbar puncture was performed, and a cerebrospinal fluid sample was sent for viral PCR as well as the usual routine tests. Intravenous acyclovir was started empirically to address the possibility of viral encephalitis.

**Figure 1:**
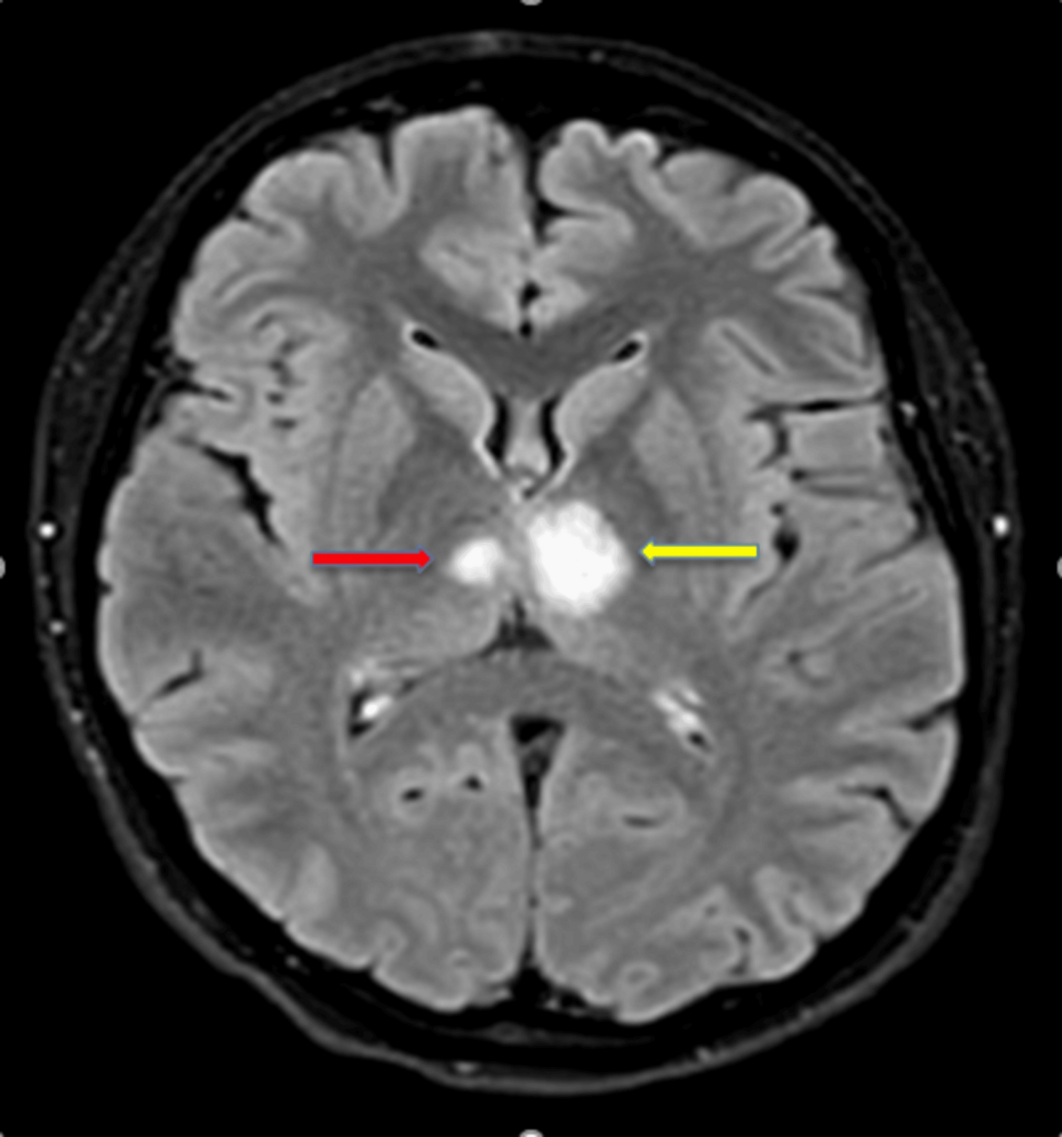
Fluid-attenuated inversion recovery (FLAIR) imaging showing two sizable foci of hyperintense lesions in the bilateral thalami. The lesion on the right measured 12 mm (red arrow), and the lesion on the left measured 22 mm (yellow arrow). These lesions were also noted in T2-weighted imaging and diffusion restriction on diffusion-weighted imaging (DWI). These features are consistent with acute infarcts.

**Figure 2:**
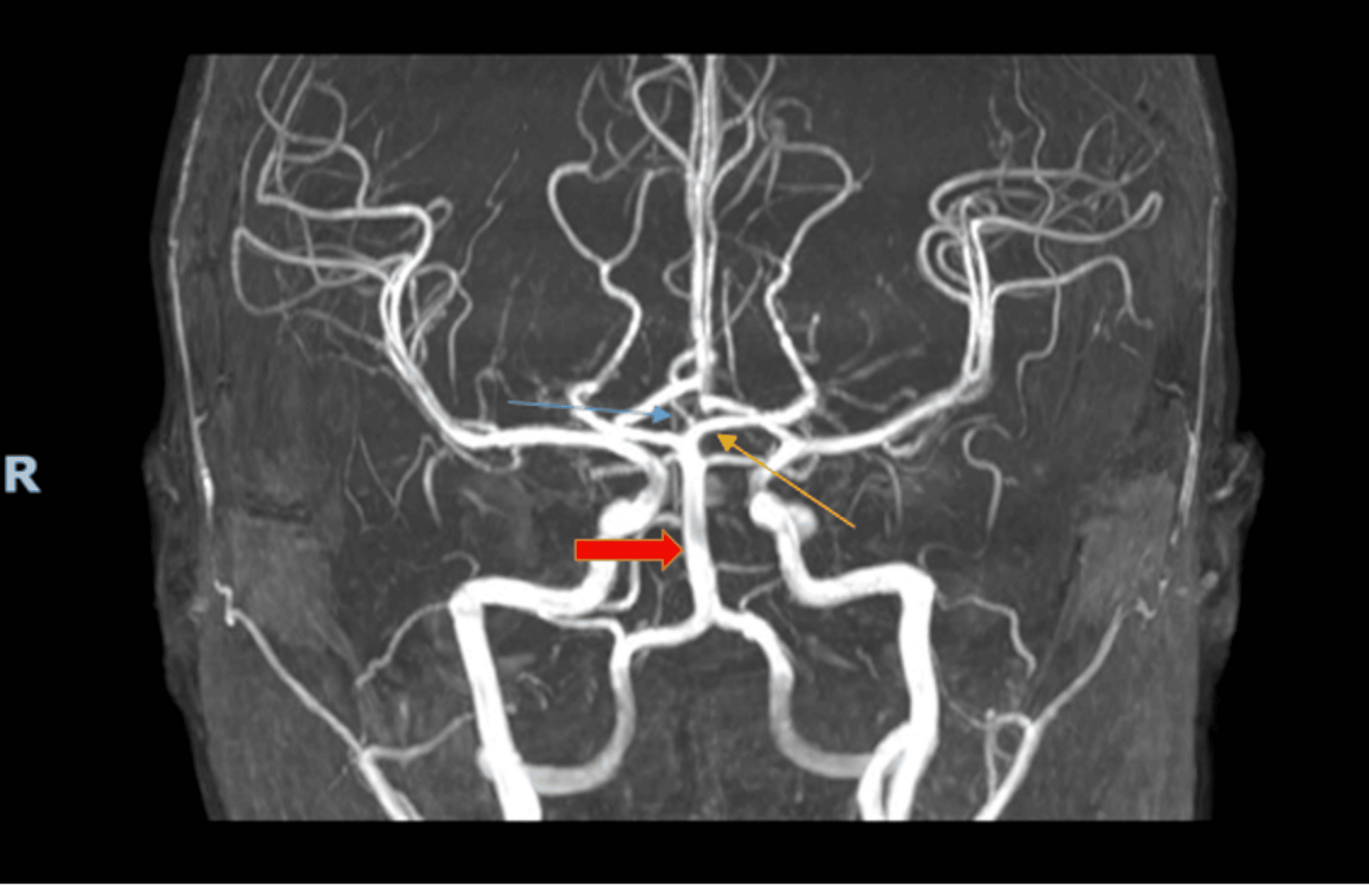
Magnetic resonance angiogram revealing a left-sided artery of Percheron (blue arrow). The thick red arrow points to the basilar artery, and the thin yellow arrow points to the left posterior cerebral artery.

MRI of the head on the following day revealed bilateral thalamic infarcts (Figure 1), and MRA identified a left-sided AOP. These results led to the conclusion that occlusion of the AOP was the cause of the stroke that the patient had suffered (Figure 2).

After reviewing the above results, acyclovir was stopped, and the patient was started on aspirin 300 mg and atorvastatin 40 mg for prophylaxis of secondary ischemic stroke. In view of her cognitive impairment, the patient was given specialized cognitive therapy to help support her recovery and increase her chances of returning to work as a veterinarian.

Further post-stroke investigations included a 14-day cardiac tape, which revealed no evidence of atrial fibrillation or any other embolic arrhythmias. In addition, young stroke blood screen tests ruled out the possibility of connective tissue disease, vasculitis, antiphospholipid syndrome, homocystinuria, JAK-2 mutation, or paroxysmal nocturnal hemoglobinuria.

Further testing with a transthoracic echocardiography (TTE) demonstrated normal left ventricular size and wall thickness with preserved systolic function (estimated ejection fraction of 60-65%). The right ventricle was normal in size and function, with no echocardiographic evidence of pulmonary hypertension. The atria were non-dilated, and no significant valvular abnormalities were identified. Although no definite interatrial shunt was visualized on color Doppler, a shunt could not be excluded on TTE alone.

A contrast (“bubble”) echocardiogram was therefore performed, which revealed the early passage of >50 microbubbles into the left atrium within the first one to two cardiac cycles, consistent with a large right-to-left interatrial shunt, most likely a PFO.

The patient’s risk of paradoxical embolism score of 10 indicated an 88% chance that her stroke was due to PFO and a 2% risk of recurrence of stroke or transient ischemic attack within two years. Her PFO-Associated Stroke Causal Likelihood classification indicated that a probable-related stroke was likely. Given these findings, a cardiology consultation was obtained to consider percutaneous closure of the PFO. Anticoagulation was instituted while awaiting the final management decision.

The patient improved and was able to return home with family support three days after admission, and was advised to resume work in two months.

## Discussion

Occlusion of the AOP

Bilateral thalamic infarctions are uncommon and often pose a diagnostic challenge because of their non-specific and variable clinical manifestations [1]. The thalamus plays a central role in consciousness, cognition, and sensory integration, and its involvement can result in altered mental status, memory impairment, and ocular motor disturbances that mimic infectious, metabolic, and/or toxic encephalopathies [1].

Among the vascular causes, the AOP, a solitary perforating vessel arising from the posterior cerebral artery (PCA), is a rare but clinically important variant that was first described by Percheron in 1973 [2]. Occlusion of this artery can lead to characteristic bilateral paramedian thalamic infarctions, sometimes extending to the rostral midbrain [3].

Epidemiology of AOP infarction

The prevalence of AOP infarction is estimated at 0.1%-2% of all ischemic strokes and 4%-18% of thalamic strokes, though the condition is likely underdiagnosed. In a study of 1,000 consecutive first-time stroke patients, isolated thalamic infarcts represented 11% of posterior circulation strokes, whereas midbrain infarctions accounted for only 7% [4].

The risk factors for AOP occlusion mirror those of general ischemic stroke. These factors include small vessel disease (33.0%-38.9%), cardioembolic sources (0%-22.0%), large vessel disease (13.2%-22.2%), and other causes such as vasospasm secondary to subarachnoid hemorrhage, hemodynamic alterations, vascular dissection, distal PCA ischemia, basilar artery aneurysms, hypercoagulable states, or CNS infection-related vasculitis (13%-15.7%), while idiopathic causes account for approximately 10% of cases [4].

Anatomy

**Figure 3:**
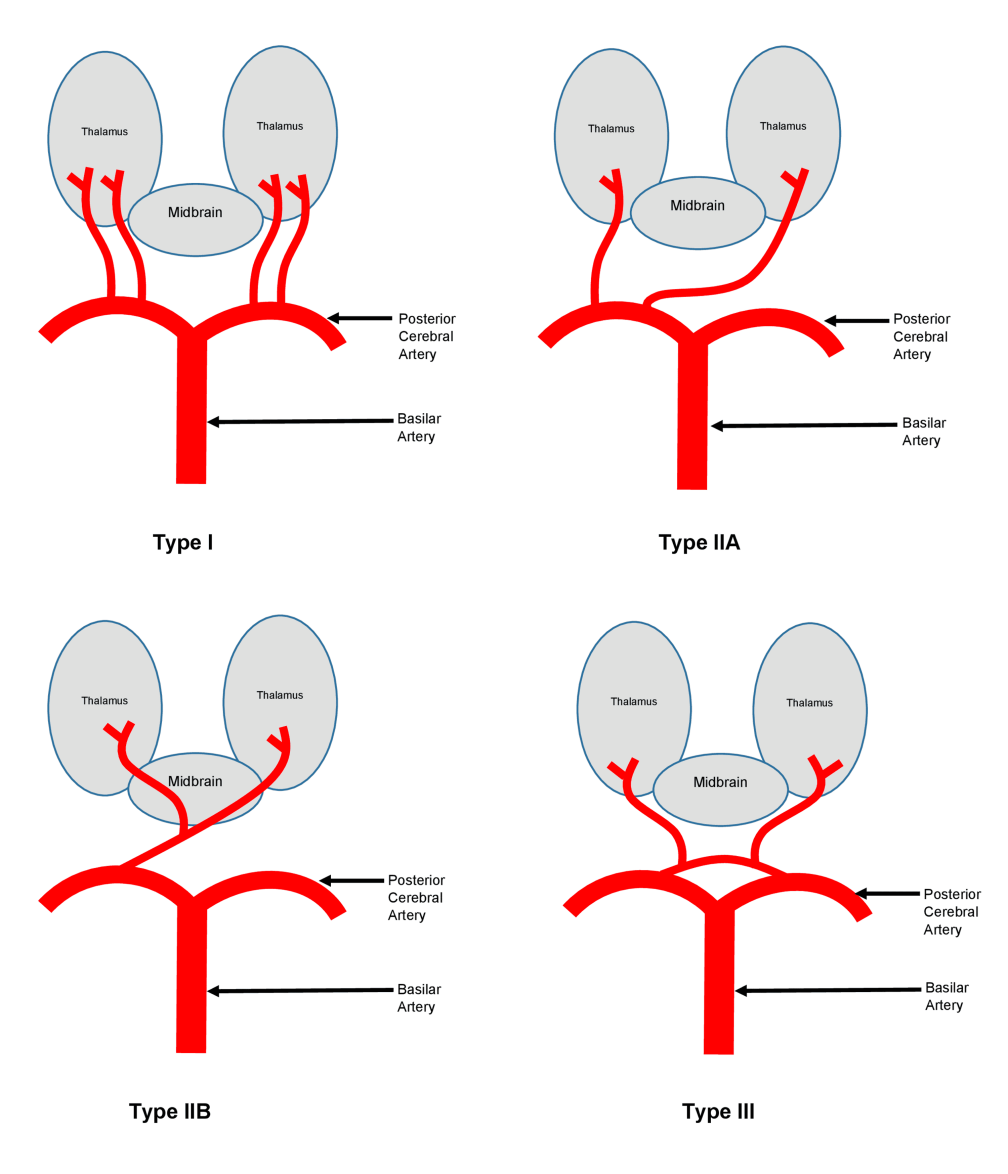
Variants of thalamic perforating arteries. This figure was created by the authors based on information from reference [5], which was published under the Creative Commons Attribution 4.0 International License.

The thalamus is normally irrigated by branches arising from the posterior communicating arteries (PcomA) and from the P1 and P2 segments of the PCA. Its vascular supply is typically divided into anterior, paramedian, inferolateral, and posterior regions. The anterior region is supplied by the polar arteries, which stem from the PcomA; the paramedian region is nourished by the thalamo-perforating arteries, which originate from the P1 segment of the PCA; the inferolateral region receives blood from the thalamo-geniculate arteries branching from the P2 segment of the PCA; and the posterior region is irrigated by the posterior choroidal arteries, which are also derived from the P2 segment. Notably, the thalamic vasculature, especially the polar artery, shows considerable anatomical variation, with as many as eight distinct configurations. In 1973, French neuropathologist Gérard Percheron [2] identified four main variants of the thalamic blood supply, including the AOP, the distinctive vessel that now bears his name. This uncommon variant consists of a single thalamic-perforating branch (Figure 3). Occlusion of this artery produces the characteristic pattern of bilateral paramedian thalamic infarction, sometimes accompanied by midbrain involvement.

Four recognized variants of the thalamic perforating arteries have been described (Figure 3). In variant I, the most common variant, symmetrical perforating arteries arise from both left and right PCAs. Variant IIa is a rare asymmetrical configuration in which perforating arteries originate exclusively from the P1 segment of one PCA. Variant IIb, which is also uncommon, is the classic AOP, which arises as a single arterial trunk from the P1 segment of one PCA and bifurcates to supply the bilateral paramedian thalami and the rostral midbrain; occlusion of this variant produces a characteristic bilateral thalamic infarction pattern. In variant III (an arcade variant), multiple small perforating branches emerge from an arterial arch connecting the P1 and P2 segments of the PCA [2].

Presentation of AOP infarcts

Clinically, AOP infarcts often present with the triad of altered mental status, vertical gaze palsy, and memory impairment, though presentation can vary widely, and symptoms such as hypersomnolence, akinetic mutism, and oculomotor dysfunction may also occur [6]. These non-specific and varied manifestations frequently lead to misdiagnosis or delayed diagnosis, particularly in younger patients without traditional vascular risk factors. The diversity of presentation is attributable to the central role of the thalamus in consciousness, cognition, and motor integration, and it underscores the importance of clinical vigilance and advanced imaging for recognizing this rare yet critical subtype of stroke.

The clinical manifestations vary according to the thalamic subregions involved and may overlap with midbrain syndromes. The classic triad of AOP infarction manifestations consists of altered mental status, memory impairment, and vertical gaze palsy [6]. Specifically, bilateral vertical gaze palsy occurs in 65% of patients, anterograde and retrograde amnesia in 58%, confusion in 53%, and coma in 42%. Onset is immediate in 40% of cases and progressive over several hours in 60% of cases. Additional neurological signs include hypersomnia, partial disorientation, akinetic mutism, speech disturbances (dysarthria, aphasia with preserved syntax but reduced verbal fluency, and occasional paraphasic errors), apraxia, dysgraphia, miosis, photophobia, asterixis, loss of convergence, and ataxic gait. Neuropsychiatric changes such as apathy and aggressiveness have also been reported. These variable and often nonspecific presentations contribute to frequent diagnostic delays and demonstrate the need for heightened clinical suspicion in the appropriate contexts [6,7].

Differential diagnosis of AOP infarction

Differentiating AOP infarction from other causes of bilateral thalamic lesions is crucial, particularly given the overlapping imaging and clinical features with conditions such as Wernicke's encephalopathy, viral encephalitis, cerebral sinus thrombosis, and metabolic disorders [8,9]. Accurate diagnosis hinges on both radiological pattern recognition and awareness of this uncommon vascular variant (Table 1).

**Table 1:** Differential diagnosis of bilateral thalamic lesions. DWI: diffusion-weighted imaging; FLAIR: fluid-attenuated inversion recovery; MRV: magnetic resonance venography; PET: positron emission tomography. This table was created by the authors based on information from references [1] and [6].

Category	Condition	Clinical features	Imaging findings
Vascular	Artery of Percheron infarct	Somnolence, memory loss, vertical gaze palsy	Bilateral paramedian thalamic ± midbrain infarcts on DWI/FLAIR
	Cerebral venous sinus thrombosis (e.g., vein of Galen)	Headache, altered consciousness, seizures	Bilateral thalamic edema, hyperintense veins, venous infarction pattern on MRV
	Top of the basilar syndrome	Coma, quadriplegia, oculomotor palsy	Infarcts in the thalami, midbrain, and occipital lobes
Infectious	Japanese encephalitis	Fever, confusion, seizures, focal deficits	Bilateral thalamic hyperintensities with edema
	West Nile virus encephalitis	Flu-like symptoms, confusion, tremor	T2 hyperintensity in the thalami, basal ganglia, and midbrain
	Toxoplasmosis (immunocompromised)	Fever, focal neurologic signs	Ring-enhancing lesions in deep gray matter (thalamus, basal ganglia)
Metabolic/toxic	Wernicke’s encephalopathy	Ataxia, ophthalmoplegia, confusion	Symmetric thalamic hyperintensity on T2/FLAIR; affects mammillary bodies, periaqueductal gray
	Osmotic demyelination (extrapontine)	Altered mental status after rapid Na+ correction	Bilateral T2 hyperintensities in the thalami and basal ganglia
	Wilson’s disease	Movement disorders, psychiatric symptoms	T2 hyperintensity in the thalami and basal ganglia; "face of giant panda" sign
	Leigh syndrome (mitochondrial)	Childhood onset, psychomotor regression, respiratory failure	T2 hyperintensity in the thalami, brainstem, and basal ganglia
	Gangliosidosis	Developmental delay, regression, spasticity	Symmetric white matter changes, thalamic calcification
Neoplastic	Bilateral thalamic glioma	Movement disorders, cognitive decline	Diffuse T2 hyperintensity, mass effect without contrast enhancement
Autoimmune/other	Posterior reversible encephalopathy	Seizures, visual disturbance, hypertension	Vasogenic edema in occipital and parietal lobes ± thalami
	Creutzfeldt-Jakob disease	Rapid dementia, myoclonus	Pulvinar sign; high DWI signal in the thalami and basal ganglia
	Fatal familial insomnia	Progressive insomnia, autonomic failure	Thalamic hypometabolism on PET; subtle thalamic atrophy

Diagnosis of AOP infarction

Prompt identification of AOP infarction requires a high index of suspicion and advanced imaging. Computed tomography (CT) is often normal in the acute phase, while modalities such as MRI scans with diffusion-weighted imaging (DWI) and fluid-attenuated inversion recovery (FLAIR) sequences are more sensitive to the subtle bilateral paramedian thalamic changes typical of this condition [10].

Because of its deep-seatedness and small caliber, the AOP is often invisible on conventional MRA or computed tomography angiography (CTA), thus posing a significant diagnostic challenge. In our case, DWI and FLAIR sequences confirmed bilateral thalamic infarction with a visible AOP on MRA, which is relatively uncommon given the low angiographic sensitivity reported in the literature [10].

Management of AOP infarction

Although no definitive treatment exists for AOP infarction beyond standard ischemic stroke management, early identification remains critical for timely intervention. In cases presenting within the therapeutic window, thrombolysis has been reported to improve outcomes [4,11]. However, delayed recognition resulting from the often non-focal and non-lateralizing nature of symptoms frequently results in missed opportunities for reperfusion therapies.

The management of AOP occlusion typically involves standard secondary prevention strategies for ischemic stroke, including antiplatelet therapy and lipid-lowering agents. A comprehensive diagnostic workup, which is recommended for all stroke patients, should be performed, including prolonged cardiac monitoring to detect paroxysmal atrial fibrillation and transthoracic echocardiography with bubble study to assess for cardiac sources of embolism, such as a PFO [11]. Where appropriate, further investigations, including a young stroke blood panel, should be performed to evaluate for conditions such as antiphospholipid syndrome, JAK2 mutation, or paroxysmal nocturnal hemoglobinuria.

The clinical presentation of our case aligns with prior reports of AOP infarction in young adults, where early symptoms may mimic encephalopathy rather than a focal neurological deficit. Additionally, the identification of a patent foramen ovale in this patient supports a potential paradoxical embolic mechanism, emphasizing the importance of targeted cardiac evaluation in stroke of undetermined etiology in younger patients [11].

Prognosis for AOP infarction

The prognosis for AOP infarction varies widely. While some individuals regain functional independence, others experience lasting cognitive and behavioral impairments. In one reported series, more than half of the patients showed favorable outcomes after three months, although deficits in attention, memory, and motivation were common [12].

This case presentation is consistent with previously reported cases of AOP infarction in young patients, where the initial symptoms often resemble encephalopathy rather than a focal neurological syndrome. Similar cases have identified paradoxical embolism via a patent foramen ovale as a plausible mechanism, highlighting the importance of targeted cardiac evaluation in stroke of undetermined etiology in this age group.

## Conclusions

Bilateral thalamic infarction from occlusion of the AOP is a rare posterior circulation stroke. Prompt neuroimaging and comprehensive evaluation, especially looking for cardioembolic sources in young patients, are essential. Interdisciplinary management optimizes treatment, prevents recurrence, and improves outcomes.
